# A security-aware service function chain deployment method for load balance and delay optimization

**DOI:** 10.1038/s41598-022-14494-2

**Published:** 2022-06-21

**Authors:** Dong Zhai, Xiangru Meng, Zhenhua Yu, Hang Hu, Tao Huang

**Affiliations:** 1 Information and Navigation College, Air Force Engineering University, Xi’an, 710077 China; 2 Institute of Systems Security and Control, College of Computer Science and Technology, Xi’an University of Science and Technology, Xi’an, 710054 China

**Keywords:** Engineering, Mathematics and computing

## Abstract

Network function virtualization (NFV) decouples network functions from hardware devices. However, it introduces security challenges due to its reliance on software, which facilitates attacks. This security problem has a significant negative impact on the interests of users. Existing deployment methods are not suitable for SFC requests with a security demand, causing the use of substrate resources unreasonable and lower acceptance ratio. Moreover, a strict delay requirement is another challenge for NFV. To make the use of the substrate resources more reasonable and reduce the transmission delay, this paper proposes a security-constraint and function-mutex-constraint consolidation (SFMC) method for virtual network function (VNF) to reduce resource consumption and transmission delay. In addition, a security-aware service function chain (SASFC) deployment method for load balance and delay optimization is presented, which deploys service function chains according to the consolidated results of the SFMC method. The SASFC method first obtains a candidate server node set using resource, hosting capacity, security and node load constraints. It then obtains candidate paths according to the metric of the minimum transmission delay and link load constraint using the Viterbi algorithm. Finally, the path with the highest VNF security level match degree among the candidate paths is adopted to deploy virtual links, and the corresponding server nodes are employed to deploy VNFs. As a result, the SASFC method makes the use of substrate resources more reasonable. It improves the acceptance ratio and long-term average revenue to cost ratio, reduces transmission delay, and achieves load balancing. Experiment results show that when the number of VNFs is five, the acceptance ratio and long-term average revenue to cost ratio of the SASFC method are close to 0.75 and 0.88, which are higher than those of the compared methods. Its transmission delay and proportion of bottleneck nodes are 7.71 and 0.024, which are lower than those of the compared methods. The simulations demonstrate the effectiveness of the SASFC method.

## Introduction

**Figure 1:**
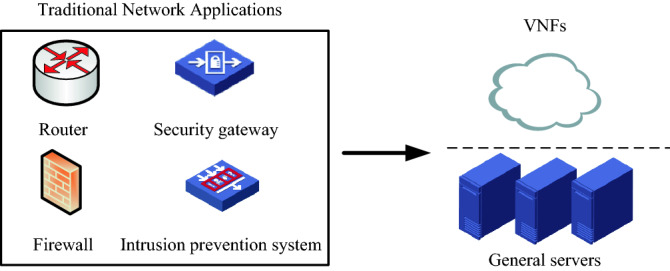
Network function virtualization scheme.

Network function and hardware are tightly coupled in traditional network. Network services require specialized hardware modules, which increase the difficulties associated with scaling and network management, and result in high resource consumption^1,2^. There is a constant increase in network congestion and delay due to the hundreds of millions of smart connected devices and an increase in mobile data traffic. To solve the abovementioned problems, global telecom companies (e.g., AT&T and BT) have directed focus toward network function virtualization (NFV). As show in Fig. 1, NFV decouples network functions and hardware devices, and implements services by running virtual network functions (VNFs) in a required order as service function chains (SFCs)^3,4^. Moreover, NFV runs VNF instances on commodity servers (e.g., × 86 servers) instead of dedicated hardware devices, which can provide flexibility, agility and dynamic management of networks, and significantly reduce resource consumption^5,6^.

It should be noted that NFV represents a breakthrough in the field of networks, however, it also introduces security risks due to its reliance on software, thus facilitating attacks^7,8^. In particular, if a potential attacker attacks a server, it can easily attack the VNFs deployed on the server. In addition, an attacker can launch a bypass attack (e.g., denial-of-service attack) to disable networks. Furthermore, an attacker may uniquely attacks substrate links (e.g., replay attacks). The access to a public infrastructure for a multi-tenant network based on NFV inherently allows for additional security risks due to the shared resources between virtual machines. These security risks may violate the information confidentiality and integrity, and impede the large-scale application of NFV. Security requirements for several services (e.g., financial services) are critical. For example, if the security requirements of bank services cannot be satisfied, there are significant losses to individuals and society. Therefore, it is challenging to satisfy the security service level agreement (SSLA) of services. This study assumes that the server nodes, substrate links, and VNFs have a security level that can defend against potential attacks (e.g., VNF attacks on server nodes, server node attacks on VNFs, user attacks on VNFs, user attacks on server nodes and substrate links).

The security level of a server node quantifies how much protection mechanisms it can provide for VNFs. The security demand of a server node quantifies how much security assurances it needs to defend attacks. The security level of a VNF quantifies how much protection mechanisms it can provide for server nodes or other VNFs. The security demand of a VNF quantifies how much security assurances it needs to defend attacks^9–11^. The deployment of SFCs with security requirements should satisfy the security constraints, which are as follows. (1) The security level of a server node should be equal to or greater than the security demand levels of VNFs deployed on it; (2) the security level of a VNF should be equal to or greater than the security demand level of the server node hosting it; (3) the security level of a VNF should be equal to or greater than the security demand levels of VNFs co-deployed on the server node with the first VNF; and (4) the security level of a substrate link should be equal to or greater than the security demand levels of the virtual links deployed on it. The security constraints are different from resource constraints; thus, the problem associated with SFC deployment is more complex.

There are many works about optimal deployment of SFC^12–14^. To reduce the delay, the approach^12^ adopts the genetic algorithm to reduce the scheduling time of VNFs. To reduce deployment costs, the approach^13^ adopts Markov approximation and matching theoretic to save energy. The proposed heuristic method^14^ uses the Monte Carlo Tree Search algorithm to improve energy efficiency. However, these methods are not suitable for SFC requests with a security demand, causing the use of substrate resources unreasonable and lower acceptance ratio. Several studies are conducted on the security deployment of virtual networks. It is assumed that substrate and virtual nodes have different security demand levels and security levels^10,15^. Substrate links have different security levels, and virtual links have different security demand levels. The approach^10^ evaluates the importance of substrate nodes using the information entropy Technique for Order of Preference by Similarity to Ideal Solution (TOPSIS) algorithm, and selects appropriate substrate nodes to deploy virtual nodes according to the evaluation result. However, the metrics adopted by the information entropy TOPSIS method do not include the security demand and security levels of substrate nodes and virtual nodes. Liu et al.^16^ propose a virtual node deployment function considering the security attributes of virtual and substrate nodes. However, they assume that all virtual nodes of a virtual network request have the same security attributes, and do not consider the security attributes of virtual links. The approach^17^ considers the security attributes of virtual and substrate nodes, and applies reinforcement learning and shortest path algorithm to node and link embedding stage, respectively.

Nevertheless, few studies are conducted on the deployment problem of SFCs with security requirements. The work^18^ categorises security threats faced by NFV as network function-specific threats and general virtualization threats, and discusses these threats in detail. Fysarakis et al.^19^ propose a new framework that enhances the security of SFCs. The work^20^ proposes a blockchain-based system called BSec-NFVO that offers secure services for all operations. In addition, Rashidi et al.^21^ propose a distributed denial of service (DDoS) defense mechanism that shares resources among multiple users to alleviate DDoS attacks. The work^22^ reduces the security attacks through optimizing the virtual machine placement. To reduce the deployment cost and satisfy the SSLA, Zhao et al.^23^ propose a minimal-cost and SSLA-guaranteed SFC deployment method with feedback adjustment (MCSG-FA). The MCSG-FA method first obtains a deployment result using the maximal-security deployment method, to improve the probability of successful deployment. Thereafter, it searches other deployment results according to the metric of the minimal deployment cost. If a new deployment result satisfies the SSLA with a lower deployment cost than the first result, the new result is used. However, these methods do not fully consider the security demand levels and security levels of VNFs, virtual links, substrate nodes and substrate links. To a certain extent, these methods cause the use of substrate resources unreasonable, and reduce the acceptance ratio.

Special 5G vertical industries (e.g., Industry 4.0) have ultra-strict delay requirements (e.g., less than 1 ms in several cases)^24^. It should be noted that NFV is a key 5G technology, therefore, it has strict delay requirements. In most previous studies, VNFs and virtual links are deployed separately, which increase the length of the deployed paths, thus increasing the transmission delay^25,26^.

**Figure 2:**
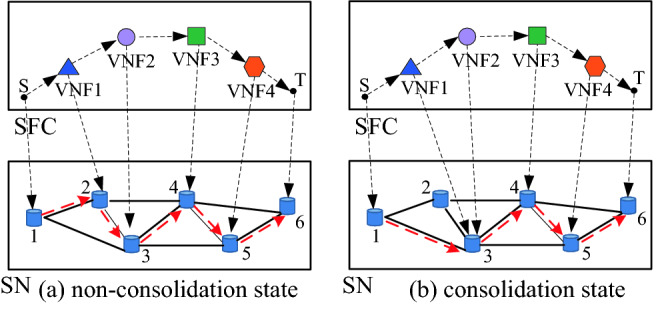
Comparison between consolidated and non-consolidated states.

In several studies, it is assumed that a server can host more than one VNF from different SFCs, however, it can host only one VNF from the same SFC^27,28^. Adjacent VNFs of an SFC on a server are consolidated according to constraints^29–31^. The consolidation of VNFs can reduce the transmission delay and bandwidth consumption. In this study, to simplify the analysis, it is assumed that the transmission delay of each hop is the same. As shown in Fig. 2, it is assumed that VNF2 and VNF3 satisfy the function mutex constraint, the security demand level of VNF2 is less than the security level of VNF3 and the security level of server node3, and the security demand level of VNF3 is less than the security level of VNF2 and the security level of server node3. Moreover, the security demand level of server node3 is less than the security level of VNF2 and the security level of VNF3. In addition, the available resources of server node3 are greater than the sum of resource demands of VNF2 and VNF3. Figure 2a presents the deployment result under the condition of non-consolidation. The hop of the entire deployment path is five, and its transmission delay is five time units. Figure 2b presents the deployment result under the condition of consolidation. The hop of the entire deployment path is four, and its transmission delay is four time units. That indicates that consolidation can effectively reduce the transmission delay.

As the problem of load imbalance is not considered^29–31^, the approach^5^ designs the optimal selection factor to achieve load balance of substrate nodes. However, the approach^5^ does not fully solve the load imbalance problem of substrate links. To make the use of the substrate resources more reasonable, reduce the transmission delay, and achieve load balance, this paper proposes the security-constraint and function-mutex-constraint consolidation (SFMC) method, and security-aware service function chain (SASFC) deployment method for load balance and delay optimization.

This paper mainly studies the deployment for SFC requests with security requirement, and does not consider cyber attacks.

The contributions of this study are as follows. (i) We model the SFC deployment problem with a security demand using integer linear programming (ILP). (ii) We present a security-constraint and function-mutex-constraint consolidation (SFMC) method that consolidates VNFs to reduce resource consumption and improve the acceptance ratio. (iii) We present a security-aware service function chain (SASFC) deployment method for load balance and delay optimization. The SASFC method uses Viterbi algorithm to jointly deploy VNFs and virtual links according to the consolidated result of the SFMC method. Therefore, it effectively reduces transmission delay and resource consumption.

## Problem statement, network model and method

### Problem statement

For SFC requests with a security demand, the first objective of deployment is to improve the acceptance ratio. The second objective is to reduce transmission delay, and the third is to achieve load balancing. The deployment of SFCs should satisfy the SSLA due to the security requirements of network services. Servers with different security levels have different charges. Several companies (e.g., Huawei and Google) generally provide different security-level severs that users can select from. To better handle the security challenges of SFCs, it is assumed that each server node and substrate link has a security level that can defend against attacks^9,16^. Simultaneously, we abstract the security attributes of SFCs, and assign different security demand levels to different VNFs and virtual links.

### Network model

A substrate network (SN), modeled as a weighted undirected graph $G_{s} = (V_{s} ,E_{s} )$, is composed of substrate nodes and links. Substrate nodes are composed of server and switch nodes. The substrate node set, as denoted by *V*_*s*_, is defined as $V_{s} = \left\{ {v_{i} \left| {i = 1,2} \right., \ldots ,\left| {V_{s} } \right|} \right\}$. The server node set, as denoted by *V*_*s,s*_, is defined as $V_{s,s} = \left\{ {v_{s,i} \left| {i = 1,2} \right., \ldots ,\left| {V_{s,s} } \right|} \right\}$. A server node *v*_*s,i*_ has the following attributes: available CPU resources *C*(*v*_*s,i*_), security level *Sl*(*v*_*s,i*_), security demand level *Sdl*(*v*_*s,i*_), and the hosting capacity. The real-time load of the server node *v*_*s,i*_ is denoted by the notation *N*_*load*_(*v*_*s,i*_). The substrate link set, as denoted by *E*_*s*_, is defined as $E_{s} = \left\{ {e_{i} \left| {i = 1,2} \right., \ldots ,\left| {E_{s} } \right|} \right\}$. For a substrate link *e*_*i*_, it has the following attributes: available bandwidth resources *B*(*e*_*i*_) and security level *Sl*(*e*_*i*_). The real-time load of the substrate link *e*_*i*_ is denoted by the notation *N*_*load*_(*e*_*i*_). The notations |*V*_*s*_|, |*V*_*s,s*_|, and |*E*_*s*_| represent the number of substrate nodes, server nodes and substrate links, respectively. The substrate link between server nodes *v*_*s,i*_ and *v*_*s,j*_ is represented by the notation *e*_*i,j*_. The notation *h*(*e*_*i,j*_) represents the hop of the substrate link *e*_*i,j*_.

Service function chain (SFC) requests consist of multiple VNFs and virtual links. The SFC(*g*) denotes the *g*-th SFC. It is modeled as a directed graph $G_{g} = \left\{ {N_{g} ,L_{g} ,S_{g} ,T_{g} } \right\}$. The VNF set, as denoted by *N*_*g*_, is defined as $N_{g} { = }\left\{ {f_{j} \left| {j = 1,2} \right., \ldots ,\left| {N_{g} } \right|} \right\}$. For a VNF *f*_*j*_, it has the following attributes: CPU resource demand *C*(*f*_*j*_), security level *Sl*(*f*_*j*_), and security demand level *Sdl*(*f*_*j*_). The virtual link set, denoted by *L*_*g*_, is defined as $L_{g} = \left\{ {l_{j} \left| {j = 1,2} \right., \ldots ,\left| {L_{g} } \right|} \right\}$. For a virtual link *l*_*j*_, it has the following attributes: bandwidth demand *Bd*(*l*_*j*_) and security demand level *Sdl*(*l*_*j*_). The notations |*N*_*g*_| and |*L*_*g*_| represent the number of VNFs and virtual links, respectively. The notations *S*_*g*_ and *T*_*g*_ represent the source and terminal nodes of SFC(*g*), respectively. The notations *v*_*S*_ and *v*_*T*_ represent the substrate nodes that *S*_*g*_ and *T*_*g*_ are deployed on. The virtual link between VNFs *f*_*i*_ and *f*_*j*_ is represented by the notation *l*_*i,j*_.

As shown in Fig. 2, consolidating VNFs can effectively reduce transmission delay and bandwidth consumption. However, owing to restrictions or conflicts between functions, some VNFs cannot be consolidated on the same server node^31^. This is called a function mutex constraint. If VNF *f*_*i*_ of the SFC(*g*) can be consolidated with VNF *f*_*j*_ of SFC(*g*), $m_{i,j}^{g} { = }1$; otherwise, $m_{i,j}^{g} { = 0}$. When two VNFs are consolidated, the security demand level of the consolidation is equal to the larger of the security demand level of the two VNFs. The security level of the consolidation is equal to the smaller of the security level of the two VNFs. Different VNF instances have location constraints during deployment, and different operators own different licenses^32^. Therefore, a server can only host several types of VNFs. This is referred to as a hosting capacity constraint. If server node *v*_*s,i*_ can host instances of VNF *f*_*j*_, $x(v_{s,i} ,f_{j} ){ = }1$; otherwise, $x(v_{s,i} ,f_{j} ){ = 0}$.

The deployment of VNFs should satisfy the resource, function mutex, hosting capacity, and security constraints. Moreover, the deployment of virtual links should satisfy the resource and security constraints. There are several security risks for SFCs^9,10^. First, servers attack the VNFs deployed on them. Servers provide resources for VNFs under certain service level agreements. A malicious attacker in control of a server can change all aspects of the VNFs deployed on the server, including the monitoring or snooping traffic associated to the VNFs. Servers supervise hosted VNFs, and the VNFs cannot defend against attacks from the servers. Second, VNFs attack the servers hosting them. A malicious VNF can access the vulnerabilities of the server hosting it via the allocated resources, and control the server. A malicious VNF can attack the network infrastructure to disrupt the services (e.g., DoS attack). Third, VNFs attack other VNFs co-deployed on the same server, which share the same resources of the server. A malicious VNF can take advantage of the shared resources to access the vulnerabilities of other VNFs deployed on the same server, and then attack. In addition, a malicious attacker can access virtual links through the substrate links hosting them.

All the security constraints considered in this study are as follows. (1) The security level of a server node should be equal to or greater than the security demand levels of the VNFs deployed on the server node. (2) The security level of a VNF should be equal to or greater than the security demand level of the server node hosting the VNF. (3) The security level of a VNF should be equal to or greater than the security demand levels of the VNFs co-deployed on the same server node with the first VNF. (4) The security level of a substrate link should be equal to or greater than the security demand levels of the virtual links deployed on the substrate link.

**Figure 3:**
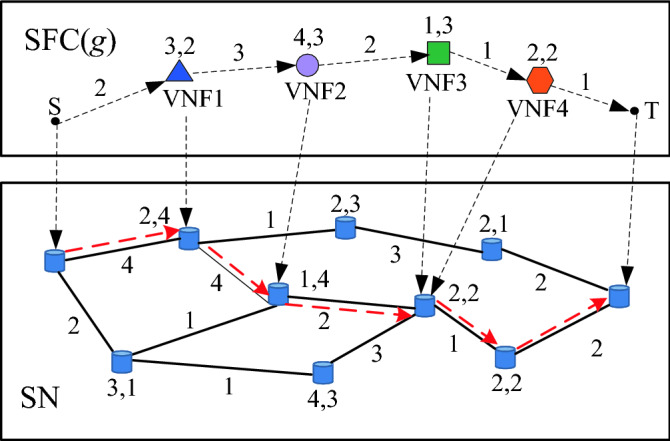
The SFC(*g*) deployment.

Figure 3 presents the deployment result of the SFC(*g*). For each server node, the three figures aside it represent its serial number, security demand level *Sdl*(*v*_*s*_) and security level *Sl*(*v*_*s*_), respectively. For each substrate link, the figure beside it represents its security level *Sl*(*e*). For each VNF, the two figures beside it represent its security demand level *Sdl*(*f*) and security level *Sl*(*f*), respectively. For each virtual link, the figure beside it represents its security demand level *Sdl*(*l*). Moreover, VNFs 3 and 4 satisfy the third security and function mutex constraints, and can therefore be deployed on the same server node.

The evaluation indicators adopted in this study are as follows. The acceptance ratio is expressed as Eq. (1)

1 $$\omega = \lim_{T \to \infty } \frac{{\sum\nolimits_{t = 0}^{T} {\left| {SFC_{deploy} (t)} \right|} }}{{\sum\nolimits_{t = 0}^{T} {\left| {SFC(t)} \right| + \delta } }}$$

where |*SFC*_*deploy*_(*t*)| and |*SFC*(*t*)| denote the number of successfully deployed SFC requests and total SFC requests at time *t*, respectively, and the notation *δ* is infinitely close to 0.

The revenue, cost, and long-term average revenue to cost ratio of the SFC(*g*) are defined as Eqs. (2), (3), and (4), respectively.

2 $$Re{(}G_{g} ,{t}{)}{ = }\sum\limits_{{f_{i} \in N_{g} }} {Sdl(f_{i} )C\left( {f_{i} } \right)} { + }\sum\limits_{{l_{j} \in L_{g} }} {Sdl(l_{j} )Bd\left( {l_{j} } \right)}$$

3 $$Co(G_{g} ,{t}{)}{ = }\sum\limits_{{v_{s,i} \in V_{s,s} }} {\sum\limits_{{f_{j} \in N_{g} }} {y_{i,j}^{g} Sl(v_{s,i} )} } C(f_{j} ) + \sum\limits_{{e_{i} \in E_{s} }} {\sum\limits_{{l_{j} \in L_{g} }} {z_{i,j}^{g} Sl(e_{i} )} } h(e_{i} )Bd(l_{j} )$$

4 $$Re/Co = \lim_{T \to \infty } \frac{{\sum\nolimits_{t = 0}^{T} {\sum\nolimits_{{G_{g} \subset SFC_{deploy} (t)}}^{{}} {Re(G_{g} ,t)} } }}{{\sum\nolimits_{t = 0}^{T} {\sum\nolimits_{{G_{g} \subset SFC_{deploy} (t)}}^{{}} {Co(G_{g} ,t)} } }}$$

where *h*(*e*_*i*_) denotes the hop of substrate link *e*_*i*_, and *SFC*_*deploy*_(*t*) denotes the set of successfully deployed SFC requests at time *t*.

The VNF security level match degree and average VNF security level match degree are expressed as Eqs. (5) and (6), respectively.

5 $$Vs(g,t) = \frac{{\sum\nolimits_{{f_{j} \in N_{g} }} {Sdl(f_{j} )} }}{{\sum\nolimits_{{v_{s,i} \in V_{s,s} }} {y_{i,j}^{g} Sl(v_{s,i} )} }}$$

6 $$AVs = \lim_{T \to \infty } \frac{{\sum\nolimits_{t = 0}^{T} {\sum\nolimits_{{G_{g} \subset SFC_{deploy} (t)}} {Vs(g,t)} } }}{{\sum\nolimits_{t = 0}^{T} {\left| {SFC_{deploy} (t)} \right|} }}$$

The link expansion coefficient is determined by the hop of the entire deployment path and the hop of the SFC(*g*), as expressed by Eq. (7). The average link expansion coefficient is expressed by Eq. (8).

7 $$K_{g} = \frac{{\sum\nolimits_{{e_{i} \in D_{g} }} {h(e_{i} )} }}{{h_{g} }} - 1$$

8 $$AK = \frac{{\sum\nolimits_{g = 1}^{{NUM_{suc} }} {K_{g} } }}{{NUM_{suc} }}$$

where the notation *D*_*g*_ represents the substrate link set that hosts the virtual links of the SFC(*g*). The notation *h*_*g*_ represents the hop of the SFC(*g*), and the notation *NUM*_*suc*_ represents the number of SFCs successfully deployed.

The average transmission delay is defined as Eq. (9).

9 $$Ade = \frac{{\sum\nolimits_{g = 1}^{{NUM_{suc} }} {de_{g} } }}{{NUM_{suc} }}$$

where the notation *de*_*g*_ represents the transmission delay of the SFC(*g*).

If the load on server nodes (or substrate links) exceeds 95%, the server nodes (or substrate links) are defined as bottleneck nodes (or links). The proportion of bottleneck nodes and links can be expressed as Eqs. (10) and (11).

10 $$Pbn = \frac{{V_{nodeload - 0.95} }}{{\left| {V_{s,s} } \right|}}$$

11 $$Pbl = \frac{{E_{nodeload - 0.95} }}{{\left| {E_{s} } \right|}}$$

where the notations $V_{nodeload - 0.95}$ and $E_{nodeload - 0.95}$ represent the numbers of bottleneck nodes and links, respectively. The notations |*V*_*s,s*_| and |*E*_*s*_| represent the numbers of all server nodes and substrate links, respectively.

### Integer linear programming model

This study models the SFC deployment problem with a security demand as integer linear programming (ILP). The objective function is to obtain the maximum long-term average revenue to cost ratio, as follows:

12 $$\max \left\{ {\lim_{T \to \infty } \frac{{\sum\nolimits_{t = 0}^{T} {\sum\nolimits_{{G_{g} \subset SFC_{deploy} (t)}}^{{}} {Re(G_{g} ,t)} } }}{{\sum\nolimits_{t = 0}^{T} {\sum\nolimits_{{G_{g} \subset SFC_{deploy} (t)}}^{{}} {Co(G_{g} ,t)} } }}} \right\}$$

The constraints are as follows:

13 $$y_{i,j}^{g} { = }\left\{ {\begin{array}{*{20}l} 1 \hfill & {\quad {\text{if}}\;f_{j} \;{\text{is}}\;{\text{deployed on}}\;v_{s,i} } \hfill \\ 0 \hfill & {\quad {\text{otherwise}}} \hfill \\ \end{array} } \right.\quad \forall f_{j} \in N_{g} ,\;\forall v_{s,i} \in V_{s,s}$$

14 $$\sum\limits_{{v_{s,i} \in V_{s,s} }} {y_{i,j}^{g} = 1} \quad \forall f_{j} \in N_{g}$$

15 $$y_{i,j}^{g} \times C(f_{j} ) \le C(v_{s,i} )\quad \forall v_{s,i} \in V_{s,s} ,\quad \forall f_{j} \in N_{g}$$

16 $$y_{i,j}^{g} \times N_{load} (v_{s,i} ) \le 95\% \quad \forall v_{s,i} \in V_{s,s} ,\quad \forall f_{j} \in N_{g}$$

17 $$\sum\limits_{{f_{j} \in N_{g} }} {y_{i,j}^{g} \le 2} ,\quad \forall v_{s,i} \in V_{s,s}$$

18 $$\begin{aligned} & {\text{if}}\;\;\;y_{i,j}^{g} \times y_{i,j + 1}^{g} { = 1 }\;\;\;\;\forall v_{s,i} \in V_{s,s} ,\;f_{j} \in N_{g} ,\;f_{j + 1} \in N_{g} \\ & {then}\;\;\;\;\;\;\;\;y_{i,j}^{g} \times y_{i,j + 1}^{g} \times m_{j,j + 1}^{g} { = 1 } \\ \end{aligned}$$

In constraint (13), if VNF *f*_*j*_ of the SFC(g) is deployed on server node *v*_*s,i*_, $y_{i,j}^{g} { = }1$; otherwise, $y_{i,j}^{g} { = 0}$. Constraint (14) ensures that VNF *f*_*j*_ is deployed on one server node. Constraint (15) ensures that the server nodes hosting the VNFs satisfy the CPU resource constraint. The notation $N_{load} (v_{s,i} )$ denotes the real-time load of server node *v*_*s,i*_. Constraint (16) ensures that the real-time load of the server nodes hosting VNFs satisfies the node load constraint. To simplify the analysis, in this paper, it is assumed that each server node can host a maximum of two VNFs of an SFC, as expressed by constraint (17). In addition, constraint (18) ensures that the two VNFs deployed on the same server node satisfy the function mutex constraint.

19 $$z_{i,j}^{g} = \left\{ {\begin{array}{*{20}l} {1\quad {\text{if}}\;l_{j} \;{\text{is}}\;{\text{deployed}}\;{\text{on}}\;e_{i} } \hfill \\ {0\quad {\text{otherwise}}} \hfill \\ \end{array} } \right.\quad \forall e_{i} \in E_{s} ,\quad \forall l_{j} \in L_{g}$$

20 $$z_{i,j}^{g} \times B(l_{j} ) \le B(e_{i} )\quad \forall e_{i} \in E_{s} ,\quad \forall l_{j} \in L_{g}$$

21 $$z_{i,j}^{g} \times N_{load} (e_{i} ) \le 95\% \quad \forall e_{i} \in E_{s} ,\quad \forall l_{j} \in L_{g}$$

In constraint (19), if virtual link *l*_*j*_ of the SFC(g) is deployed on substrate link *e*_*i*_, $z_{i,j}^{g} { = }1$; otherwise, $z_{i,j}^{g} { = 0}$. Constraint (20) ensures that the substrate links hosting virtual links satisfy the bandwidth resource constraint. The notation *N*_*load*_(*e*_*i*_) denotes the real-time load of substrate link *e*_*i*_. Constraint (21) ensures that the real-time load of the substrate links hosting virtual links satisfies the link load constraint.

22 $$y_{i,j}^{g} \times Sdl(f_{j} ) \le Sl(v_{s,i} )\quad \forall v_{s,i} \in V_{s,s} ,\quad \forall f_{j} \in N_{g}$$

23 $$y_{i,j}^{g} \times Sdl(v_{s,i} ) \le Sl(f_{j} )\quad \forall v_{s,i} \in V_{s,s} ,\quad \forall f_{j} \in N_{g}$$

24 $$y_{i,j}^{g} \times Sdl(f_{j} ) \le \min_{{f_{k} \in \Omega (v_{s,i} )}} Sl(f_{k} )\quad \forall v_{s,i} \in V_{s,s} ,\quad \forall f_{j} \in N_{g}$$

25 $$z_{i,j}^{g} \times Sdl(l_{j} ) \le Sl(e_{i} )\quad \forall e_{i} \in E_{s} ,\quad \forall l_{j} \in L_{g}$$

Constraints (22) and (23) ensure that server nodes and VNFs satisfy the first and second security constraints, respectively. The notation $\Omega (v_{s,i} )$ represents the set of VNFs deployed on server node *v*_*s,i*_. Constraint (24) ensures that VNFs satisfy the third security constraint. In addition, constraint (25) ensures that deployed links satisfy the fourth security constraint.

26 $$\sum\limits_{{v_{s,i} \in V_{s,s} }} {(y_{i,j}^{g} \times x(v_{s,i} ,f_{j} )) = 1} ,\quad \forall f_{j} \in N_{g}$$

Constraint (26) ensures that the server node hosting VNF *f*_*j*_ satisfies the hosting capacity constraint.

### Heuristic method

Since the problem of finding the optimal deployment for SFCs is NP-Hard^23,33^, the complexity of the ILP solution is significantly high. Therefore, this paper proposes the SFMC and SASFC methods to obtain a solution. The consolidation of VNFs can effectively reduce the transmission delay. Hence, this paper proposes a security-constraint and function-mutex-constraint consolidation (SFMC) method for VNFs. Deploying VNFs and virtual links separately generally results in sub-optimal deployment results. However, deploying VNFs and virtual links simultaneously would make the problem particularly complex. The Viterbi algorithm demonstrates a superior performance in dynamic programming, which can effectively reduce the problem complexity arising from the simultaneous deployment of VNFs and virtual links. With an increase in the match degree of the VNF security level, the more reasonable the deployment result is. Therefore, the SASFC method considers the VNF security level match degree, and adopts the Viterbi algorithm to simultaneously deploy VNFs and virtual links according to the consolidation results of the SFMC method. If the deployment fails, the SASFC method simultaneously deploys VNFs and virtual links according to the non-consolidation results.

The pseudocode of the SFMC method is shown in Algorithm 1. If VNFs *f*_*i*_ and *f*_*i*+1_ of the SFC(g) satisfy the function mutex and security constraints, they are consolidated, and $F_{{b_{1} }} { = }\left\{ {f_{{i{ + }b}} ,\;f_{{i{ + }b + 1}} } \right\}$ is obtained. The security demand level of $F_{{b_{1} }}$ is equal to ${\text{max}}\left\{ {f_{{i{ + }b}} ,\;f_{{i{ + }b + 1}} } \right\}$. The security level of $F_{{b_{1} }}$ is equal to ${\text{min}}\left\{ {f_{{i{ + }b}} ,\;f_{{i{ + }b + 1}} } \right\}$. If not, $F_{{b_{1} }} { = }f_{{i{ + }b}}$ is obtained (Lines 2–18). The security demand level difference constraint $\left| {Sdl(f_{i + b} ) - Sdl(f_{{i + b{ + }1}} )} \right| \le \alpha$ is considered to improve the VNF security level match degree and acceptance ratio, where *α* is the security demand level difference constant.
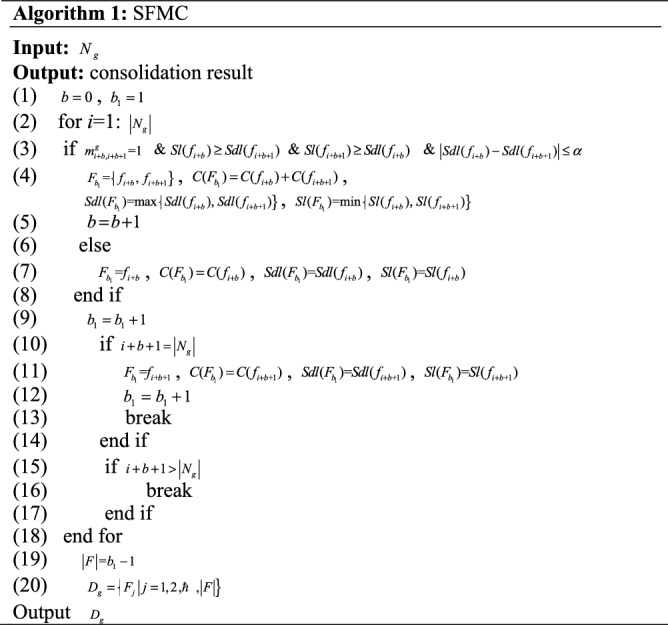


**Figure 4:**
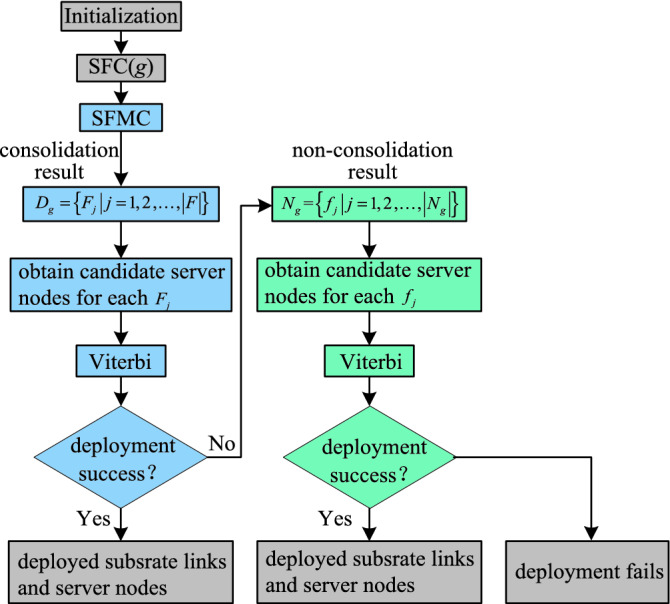
The flow chart of the SASFC method.

The flow chart of the SASFC method is shown in Fig. 4. Firstly, the SFMC method consolidates VNFs according to constraints. The SASFC method obtains candidate server node sets according to the consolidation result of the SFMC method. Thereafter it jointly deploys VNFs and virtual links using the Viterbi algorithm. The three paths with the minimal transmission delay are selected as the candidate paths, and they must satisfy the link load constraint. The SASFC method adopts the path with the highest VNF security level match degree from the candidate paths to deploy virtual links, and the corresponding server nodes are employed to deploy VNFs. If deployment fails, the SASFC method will jointly deploy VNFs and virtual links using the Viterbi algorithm according to the non-consolidation result.

**Figure 5:**
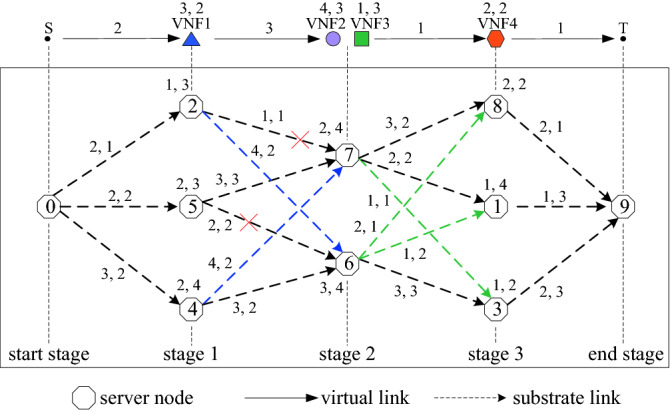
Multi-stage graph.

We assume that the SFC(*g*) is composed of four VNFs, and VNFs 2 and 3 satisfy the third security constraint and function mutex constraint; thus, they can be consolidated. Figure 5 presents the multi-stage graph for the substrate network. First, Sever nodes 0 and 9 are set as the “start” and “end” stages, respectively. All server nodes and substrate links are assumed to satisfy load constraints. Moreover, it is assumed that only Server nodes 2, 4 and 5 have more CPU resources than the CPU resource demand of VNF 1, and satisfy the security and hosting capacity constraints. Thus, Server nodes 2, 4 and 5 are selected as the candidate server nodes of VNF 1, and placed in “Stage 1”. It is assumed that only Server nodes 6 and 7 have more CPU resources than the total CPU resource demand of VNFs 2 and 3, and satisfy the security and hosting capacity constraints. Thus, Server nodes 6 and 7 are selected as the candidate server nodes simultaneously hosting VNFs 2 and 3, and placed in “Stage 2”. Furthermore, it is assumed that only Server nodes 1, 3 and 8 have more CPU resources than the CPU resource demand of VNF 4, and meet the security and hosting capacity constraints. Thus, server nodes 1, 3 and 8 are selected as the candidate server nodes of VNF 4, and placed in “Stage 3”. Each server node in one stage is connected with all server nodes of the previous and subsequent stages.

Each edge of the multi-stage graph may be composed of one or multiple substrate links. The transmission delay of the shortest path of each edge is used as its transmission delay. The two figures beside each edge denote the minimal security level and transmission delay, respectively. The two figures beside each server node denote its security attributes *Sdl*(*v*_*s*_) and *Sl*(*v*_*s*_), respectively. The two figures beside each VNF denote its security attributes *Sdl*(*f*) and *Sl*(*f*), respectively. The figure beside each virtual link denotes its security attribute *Sdl*(*l*).

The Viterbi path is computed as follows. First, for each edge between the Server node 0 and the server nodes of Stage 1, if its security level is equal to or greater than the security demand level of the corresponding virtual link, the transmission delay of this edge is recorded. If not, this edge fails. Thereafter, this process is repeated to select edges between server nodes of Stage 1 and server nodes of Stage 2. Considering Server node 7 of Stage 2 as an example, the security level of the edge between Server nodes 2 and 7 is lower than the security demand level of the corresponding virtual link; thus, this edge fails. The security levels of the edge between Server node 7 and Server node 4, and the edge between Server node 7 and Server node 5 satisfy the security constraint. Therefore, we add the transmission delay of each edge and recorded the results from the previous stage. The results are 4 and 5, respectively. We select the minimal transmission delay of 4 as the transmission delay of Server node 7, and record this result and the corresponding edges. For Server node 6, the same process is repeated.

We move from one stage to next stage until reaching “end” stage. The blue and green dotted lines represent the selected links for Stages 2 and 3, respectively. The three paths with the minimal transmission delay are selected as the candidate paths. We adopt the path with the highest VNF security level match degree (*V*_*S*_) from the candidate paths to deploy virtual links, and adopt the corresponding server nodes to deploy VNFs.

The notation *Sl* denotes the security threshold value. The notations *V*(*F*_*i*_) and *V*(*f*_*i*_) denote the candidate deployment node sets of *F*_*i*_ and *f*_*i*_, respectively. The pseudo code of the SASFC method is shown in Algorithm 2.
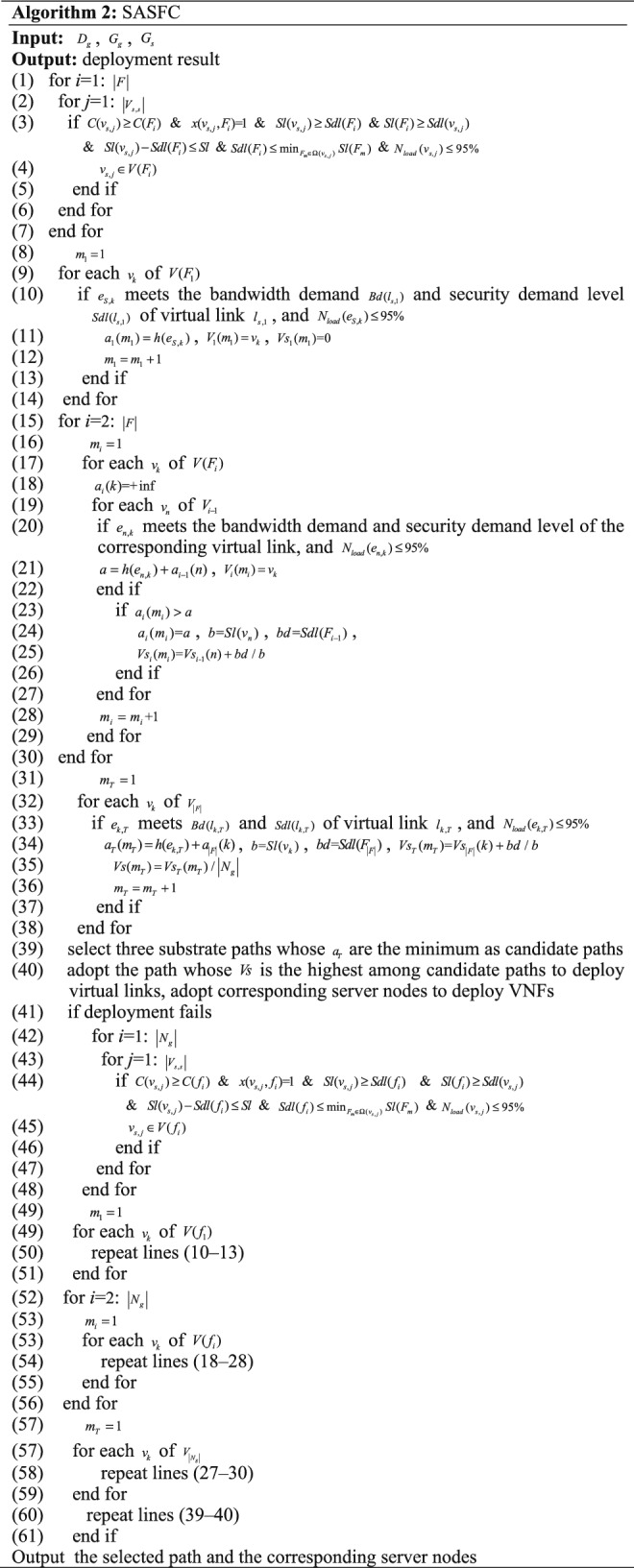


We deploy an SFC according to the consolidation result of the SFMC method. For each *F*_*i*_, we obtain a candidate server node set *V*(*F*_*i*_) according to the resource, hosting capacity, security and node load constraints (Lines 1–7). The security threshold constraint $Sl(v_{s,j} ) - Sdl(F_{i} ) \le Sl$ improves the VNF security level match degree *V*_*S*_. The security constraint $Sdl(F_{i} ) \le \min_{{F_{m} \in \Omega (v_{s,j} )}} Sl(F_{m} )$ ensures that other VNFs deployed on the same server node have a higher security level than the security demand level of *F*_*i*_. For substrate links, they should satisfy the bandwidth demand and security demand level of the corresponding virtual link. In addition, substrate links should satisfy the link load constraint $N_{load} (e_{i} ) \le 95\%$.

As shown in Fig. 5, for a server node of *V*(*F*_*i*_), we compute the transmission delays from each server node of *F*_*i*−1_ by summing up the recorded results, select the minimal transmission delay, and record this result. For other server nodes of *V*(*F*_*i*_), this process is repeated. All substrate links selected by the Viterbi algorithm should satisfy the link load constraint. We select three paths as candidate paths according to the metric of the minimal transmission delay through the Viterbi algorithm (Lines 8–39). The path with the highest *V*_*S*_ from the candidate paths is adopted to deploy virtual links, and the corresponding server nodes are adopted to deploy VNFs (Line 40).

If deployment fails, an SFC is deployed according to the non-consolidation result (Lines 41–62). For each VNF *f*_*i*_, we obtain a candidate server node set according to the resource, hosting capacity, security and node load constraints (Lines 42–48). The security threshold constraint $Sl(v_{s,j} ) - Sdl(f_{i} ) \le Sl$ improves *V*_*s*_. The process expressed by Lines 8–40 is then repeated, and the deployed path and server nodes are obtained.

### Complexity analysis

For the SFMC method, the complexity of consolidating VNFs is $O(\left| {N_{g} } \right|)$. For the SASFC method, the complexities of selecting candidate nodes and deploying SFC are $O(\left| {N_{g} } \right|\left| {V_{s,s} } \right|)$ and $O(\left| {N_{g} } \right|\left| {V_{s,s} } \right|^{2} \left| {L_{g} } \right|)$, respectively. Hence the total computational complexity for the SASFC method is $O(\left| {N_{g} } \right|\left| {V_{s,s} } \right|^{2} \left| {L_{g} } \right|)$.

## Results

### Simulation environment

The improved Salam network topology random generation algorithm is adopted to generate the substrate network topology and SFC topology. The substrate network contains 100 server nodes and switch nodes. Server and switch nodes are deployed at the same location, and different switch nodes have the 50% probability of connectivity via substrate links^30^. According to the work^34,35^, the CPU resources of server nodes and bandwidth of substrate links obey the uniform distribution of [60, 100]. There are five types of VNFs {*f*_1_, *f*_2_, *f*_3_, *f*_4_, *f*_5_}, where *f*_2_ and *f*_3_ cannot satisfy the function mutex constraint, and each server node can host any two types of the five types considered in this study. According to the work^9,10^, the security levels and security demand levels of server nodes and VNFs, security levels of substrate links, and security demand levels of virtual links obey the integer uniform distribution of [1, 4]. To simplify the analysis, it is assumed that the transmission delay of each hop for the substrate link is the same, and its value is 1 ms.

The server nodes hosting source and terminal nodes of an SFC are randomly determined according to the SFC request. The CPU resource demands of VNFs and the bandwidth demands of virtual links obey the uniform distribution of [8, 12] and [21, 24], respectively. The arrival ratio of SFC requests obeys the Poisson distribution, with a parameter of 0.05. Their duration time obeys the exponential distribution, with parameter of 1000. The security demand level difference constant *α* is set as 2, and the security threshold *Sl* is set as 2.

### Method comparison

The proposed SASFC method is compared with the MCSG-FA method^23^ and the SA-VNE method^10^ in the same experimental environment. Table 1 shows the detailed description of the three methods.

**Table 1:** Description of the three methods.

Method	Description
SASFC	The proposed method uses the Viterbi algorithm to jointly deploy VNFs and virtual links according to the consolidation result. If deployment fails, VNFs and virtual links are deployed according to the non-consolidation result
MCSG-FA	The method^23^ first obtains a deployment result through the maximal-security deployment method. Thereafter it searches other deployment results according to the metric of the minimal deployment cost. If a new deployment result meets the SSLA with lower deployment cost than the first result, the new result is used
SA-VNE	The method^10^ evaluates the importance of substrate nodes using the information entropy TOPSIS algorithm, and selects appropriate substrate nodes to deploy virtual nodes according to the evaluation result. Subsequently, it adopts the shortest path algorithm to deploy virtual links

Due to the limited research conducted on the security deployment of SFCs, we adopt the SA-VNE method as a method for comparison, which is a security deployment method for virtual networks. To conduct a more accurate comparison with the proposed algorithm, the SA-VNE method is adjusted in this study. The SA-VNE method introduces security levels of server nodes and security demand levels of VNFs into the information entropy TOPSIS algorithm. Thereafter, it evaluates the importance of server nodes using the information entropy TOPSIS algorithm and selects appropriate server nodes to host VNFs according to the evaluation results.

### Experimental results

**Figure 6:**
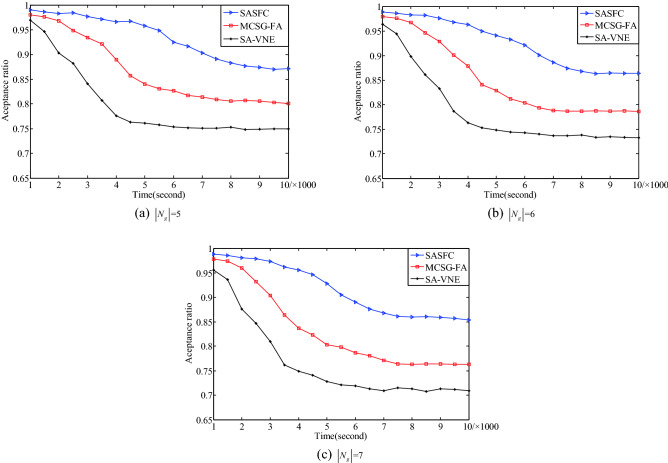
Acceptance ratio.

Figure 6 presents the experimental results of the acceptance ratio with different number of VNFs. The experimental results for five VNFs are shown in Fig. 6a. The SASFC method deploys SFCs according to the result of the SFMC method, which reduces bandwidth consumption. Moreover, the SASFC method considers the security threshold constraint and uses the Viterbi algorithm to simultaneously deploy VNFs and virtual links. Its acceptance ratio is close to 0.87. The MCSG-FA method selects the server nodes with a higher security level to obtain an initial deployment solution. Thereafter, it adjusts deployment results according to resource consumption. Therefore, its deployment results are local optimum. Its acceptance ratio is close to 0.8. The SA-VNE method evaluates the importance of server nodes using the information entropy TOPSIS algorithm. Moreover, it deploys VNFs according to the evaluation result of the information entropy TOPSIS algorithm. It adopts the shortest path algorithm to deploy virtual links. Its acceptance ratio is close to 0.75.

The experimental results for six and seven VNFs are shown in Fig. 6b, c, respectively. When the number of VNF is six, the acceptance ratios of the SASFC, MCSG-FA and SA-VNE methods are close to 0.86, 0.79 and 0.73, respectively. When the number of VNF is seven, the acceptance ratios of the SASFC, MCSG-FA and SA-VNE methods are close to 0.85, 0.76 and 0.71, respectively. The experimental results in Fig. 6 indicate that the SASFC method exhibits a higher acceptance ratio than other two methods.

**Figure 7:**
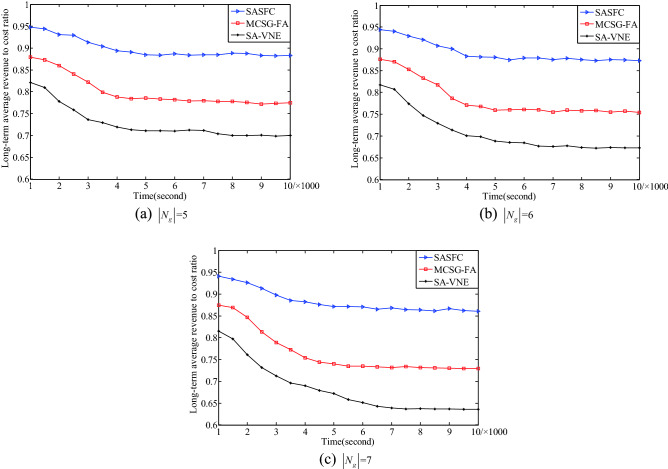
Long-term average revenue to cost ratio.

Figure 7 presents the experimental results of the long-term average revenue to cost ratio with respect to the number of VNFs. The experimental results for five VNFs are shown in Fig. 7a. The SASFC method deploys SFCs according to the consolidated results of the SFMC method. Moreover, the SASFC method considers the security threshold constraint when selecting the candidate server node set, which can improve the revenue to cost ratio. In addition, the SASFC method further reduces the bandwidth consumption by jointly deploying VNFs and virtual links. Its long-term average revenue to cost ratio is close to 0.88. The deployment results of the MCSG-FA method are local optimum. Its long-term average revenue to cost ratio is close to 0.77. The SA-VNE method deploys VNFs and virtual links separately, which would consume more bandwidth resources. Its long-term average revenue to cost ratio is close to 0.70.

The experimental results for six and seven VNFs are shown in Fig. 7b, c, respectively. When the number of VNF is six, the long-term average revenue to cost ratios of the SASFC, MCSG-FA and SA-VNE methods are close to 0.87, 0.75 and 0.67, respectively. When the number of VNF is seven, the long-term average revenue to cost ratios of the SASFC, MCSG-FA and SA-VNE methods are close to 0.86, 0.73 and 0.64, respectively. The experimental results in Fig. 7 indicate that the SASFC method exhibits a higher long-term average revenue to cost ratio than other two methods.

**Figure 8:**
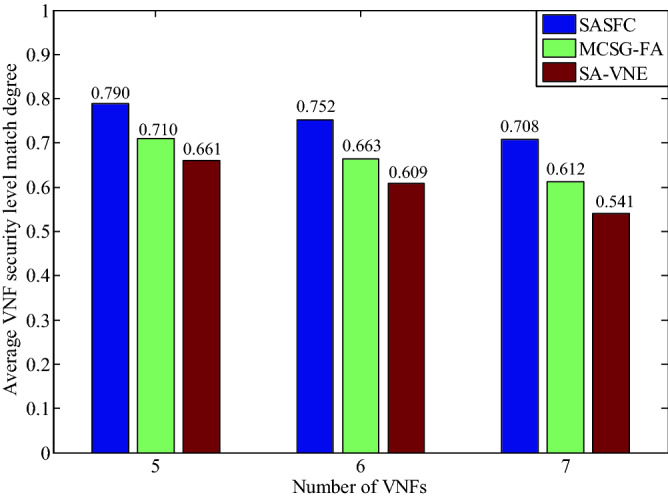
Average VNF security level match degree.

Figure 8 presents the experimental results of the average VNF security level match degree (*AVs*) with respect to the number of VNFs. The SFMC method considers the security demand level difference constraint when consolidating VNFs. The SASFC method considers the security threshold constraint when selecting a candidate server node set. The abovementioned works effectively improve the VNF security level match degree (*Vs*). In addition, the SASFC method selects the path with the highest *Vs* among the candidate paths as the deployed path. Therefore, the SASFC method exhibits the highest *AVs* among the three methods. The MCSG-FA method does not consider *Vs* when deploying SFCs. Therefore, its *AVs* is lower than that of the SASFC method. The security levels of server nodes and security demand levels of VNFs influence resource consumption. The MCSG-FA method adjusts the deployment results according to the resource consumption. Therefore, its average VNF security level match degree *AVs* is higher than that of the SA-VNE method.

**Figure 9:**
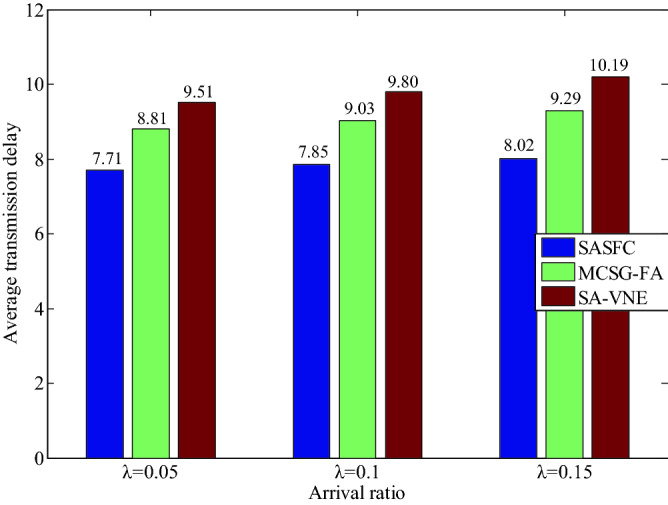
Average transmission delay.

**Figure 10:**
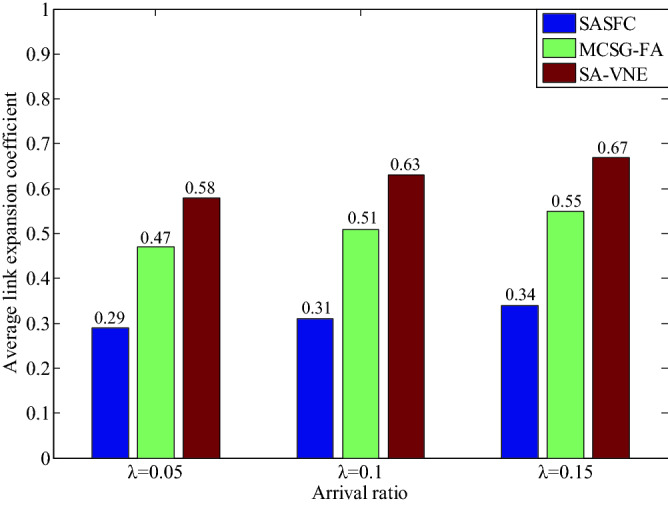
Average link expansion coefficient.

The experimental results of the average transmission delay *(Ade*) and average link expansion coefficient (*AK*) for five VNFs are shown in Figs. 9 and 10. The SASFC method adopts the Viterbi algorithm to obtain the candidate paths with the minimum transmission delay, which can effectively decrease the hop of the deployed path and transmission delay. Moreover, it deploys SFCs according to consolidated results, which effectively decreases the consumption of substrate resources. Therefore, the SASFC method exhibits a lower values of *Ade* and *AK*. As can be seen from Figs. 9 and 10, the results of *Ade* are close to 7.71, 7.85, 8.02 and the results of *AK* are close to 0.29, 0.31, 0.34, when the values of arrival ratio *λ* are 0.05, 0.1 and 0.15 respectively.

**Figure 11:**
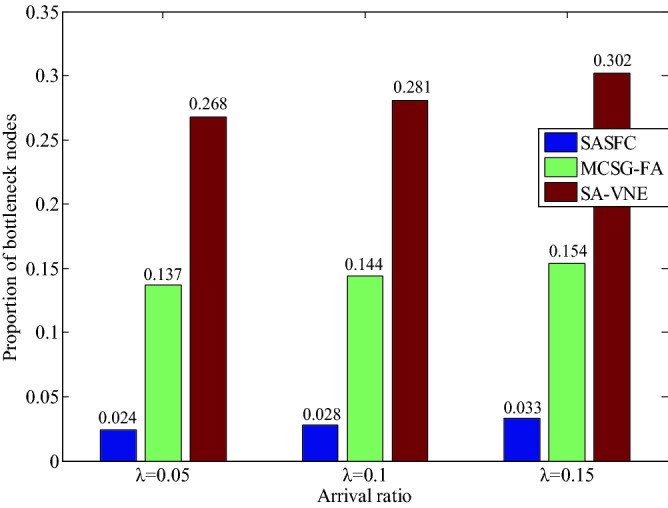
Proportion of bottleneck nodes.

**Figure 12:**
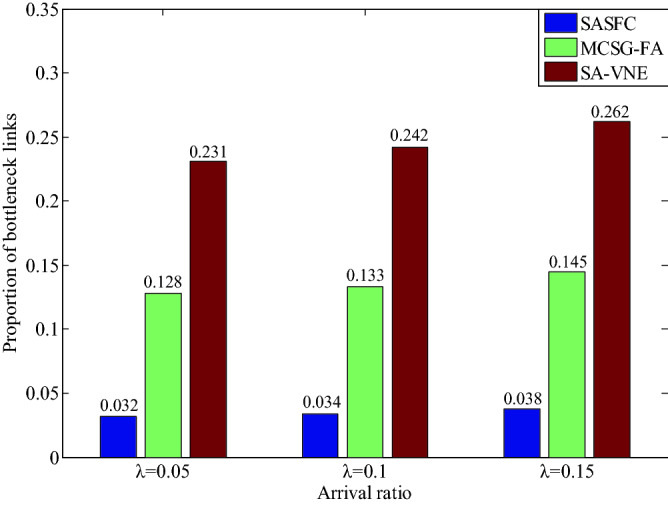
Proportion of bottleneck links.

The experimental results of the proportion of bottleneck nodes and links for five VNFs are shown in Figs. 11 and 12. As can be seen from Figs. 11 and 12, the MCSG-FA and SA-VNE methods do not fully consider the real-time load of server nodes and substrate links, thus resulting in high proportions of bottleneck nodes and links. The SASFC method selects candidate server nodes considering the node load constraint, and adopts the Viterbi algorithm to select candidate paths considering the link load constraint. As shown in Figs. 11 and 12, when the results of *λ* are equal to 0.05, 0.1 and 0.15, the proportions of bottleneck nodes of the SASFC method are 0.024, 0.028, 0.033, respectively, and the proportions of bottleneck links of the SASFC method are close to 0.032, 0.034, 0.038, respectively. The results shown in Figs. 11 and 12 verify the performance of the SASFC method in terms of load balancing.

The results in Figs. 6, 7, 8, 9, 10, 11 and 12 reveal that the SASFC method improves the acceptance ratio and average VNF security level match degree, reduces transmission delay, and achieves load balancing.

## Discussion

The SFMC method considers the security demand level difference constraint, which improves the revenue to cost ratio. The SASFC method deploys SFCs according to the results of the SFMC method, so that bandwidth consumption and transmission delay are reduced. Moreover, the SASFC method considers the security threshold constraint and the node load constraint when selecting the candidate server node set, so that it can improve the VNF security level match degree and reduce proportion of bottleneck nodes. In addition, the SASFC method uses the Viterbi algorithm to simultaneously deploy VNFs and virtual links, and considers the link load constraint when selecting candidate paths. The paths with the minimum transmission delay are selected as candidate paths through the Viterbi algorithm. Therefore, it can reduce transmission delay and proportion of bottleneck links. The SASFC method selects the path with the highest VNF security level match degree among the candidate paths as the deployed path, so that the average VNF security level match degree is improved.

The SASFC method adopts Viterbi algorithm to simultaneously deploy VNFs and virtual links, and considers security constraints, which make the use of the substrate resources more reasonable. Meanwhile, it considers the node and link load constraints, and transmission delay. Therefore, it improves the acceptance ratio, long-term average revenue to cost ratio and average VNF security level match degree, reduces the average transmission delay, proportion of bottleneck nodes, and proportion of bottleneck links.

The MCSG-FA method selects the server nodes with a higher security level to obtain an initial deployment solution. Thereafter, it adjusts deployment results according to resource consumption. Therefore, its deployment results are local optimum. The SA-VNE method evaluates the importance of server nodes using the information entropy TOPSIS algorithm. Moreover, it deploys VNFs according to the evaluation result of the information entropy TOPSIS algorithm, which may not satisfy the third security constraint. It adopts the shortest path algorithm to deploy virtual links, which may not satisfy the fourth security constraint. It deploys VNFs and virtual links separately, so that more bandwidth resources are consumed. The MCSG-FA and SA-VNE methods do not fully consider the real-time load of server nodes and substrate links. Simulation results show that the performance of the SASFC method is better than that of the MCSG-FA and SA-VNE methods.

## Conclusion

In this study, the deployment problem of SFC requests with a security demand is investigated. First, this paper proposes a security-constraint and function-mutex-constraint consolidation (SFMC) method that consolidates VNFs to reduce resource consumption and transmission delay. In addition, a security-aware service function chain (SASFC) deployment method is proposed for load balance and delay optimization.

The SASFC method deploys SFCs according to the consolidated results of the SFMC method, so that bandwidth consumption and transmission delay are reduced. Moreover it obtains a candidate server node set for VNFs through resource, hosting capacity, security and node load constraints, so that proportion of bottleneck nodes is reduced. In addition, it jointly deploys VNFs and virtual links, and obtains candidate paths using the Viterbi algorithm according to the metric of minimum transmission delay. Therefore, the transmission delay is further reduced. All substrate links selected by the Viterbi algorithm should satisfy the link load constraint. Therefore, the transmission delay and proportion of bottleneck links are reduced. The path with the highest VNF security level match degree among the candidate paths is adopted to deploy virtual links, and the corresponding server nodes are employed to deploy VNFs. As a result, the SASFC method demonstrates a higher acceptance ratio and average VNF security level match degree, and lower average transmission delay and proportion of bottleneck nodes/links than the MCSG-FA and SA-VNE methods.

Experiment results reveal that when the number of VNFs is five, the acceptance ratio and long-term average revenue to cost ratio of the SASFC method is close to 0.75 and 0.88, which are higher than that of the compared methods. Its transmission delay and proportion of bottleneck nodes are 7.71 and 0.024, which are lower than that of the compared methods. The experiment results demonstrate the effectiveness of the SASFC method.

This study mainly considers the deployment for SFC requests with security requirement. We will investigate the protective methods for special attack methods (e.g., DDoS) in the future. The proposed heuristic method can be applied to parameter estimation of COVID-19 dynamical model^36–38^.

The main notations used in this paper are listed in Table 2.

**Table 2:** Main notations.

Notations	Definitions
*G*_*s*_	Substrate network
*V*_*s*_	Set of substrate nodes
*E*_*s*_	Set of substrate links
*V*_*s,s*_	Set of server nodes
*C*(*v*_*s,i*_)	Available CPU resources of server node *v*_*s,i*_
*N*_*load*_(*v*_*s,i*_)	Real-time load of server node *v*_*s,i*_
*Sdl*(*v*_*s,i*_)	Security demand level of server node *v*_*s,i*_
*Sl*(*v*_*s,i*_)	Security level of server node *v*_*s,i*_
*B*(*e*_*j*_)	Available bandwidth of substrate link *e*_*j*_
*N*_*load*_(*e*_*j*_)	Real-time load of substrate link *e*_*j*_
*Sl*(*e*_*j*_)	Security level of substrate link *e*_*j*_
*e*_*i,j*_	Substrate link connecting server nodes *v*_*s,i*_ and *v*_*s,j*_
*h*(*e*_*i,j*_)	Hop of substrate link *e*_*i,j*_
*G*_*g*_	The *g*-th SFC
*N*_*g*_	VNF set of SFC(*g*)
*L*_*g*_	Virtual link set of SFC(*g*)
*S*_*g*_	Source node of SFC(*g*)
*T*_*g*_	Terminal node of SFC(*g*)
*C*(*f*_*j*_)	CPU resource demand of VNF *f*_*j*_
*Sdl*(*f*_*j*_)	Security demand level of VNF *f*_*j*_
*Sl*(*f*_*j*_)	Security level of VNF *f*_*j*_
*Bd*(*l*_*j*_)	Bandwidth demand of virtual link *l*_*j*_
*Sdl*(*l*_*j*_)	Security demand level of virtual link *l*_*j*_

## Data Availability

The datasets generated during and/or analyzed during the current study are available from the corresponding author on reasonable request.

## References

[1] Zhai D, Meng X, Yu Z, Han X (2021). Reliability-aware service function chain backup protection method. IEEE Access.

[2] Sun G, Xu Z, Yu H, Chang V (2021). Dynamic network function provisioning to enable network in box for industrial applications. IEEE Trans. Ind. Inf..

[3] Zhai D, Meng X, Yu Z, Hu H, Han X (2021). A fine-grained and dynamic scaling method for service function chains. Knowl. Based Syst..

[4] Mai L, Ding Y, Zhang X, Fan L, Yu S, Xu Z (2021). Energy efficiency with service availability guarantee for network function virtualization. Futur. Gener. Comput. Syst..

[5] Sun G, Xu Z, Yu H, Chen X, Chang V, Vasilakos A (2020). Low-latency and resource-efficient service function chaining orchestration in network function virtualization. IEEE Internet Things J..

[6] Ghaznavi M, Shahriar N, Kamali S, Ahmed R, Boutaba R (2017). Distributed service function chaining. IEEE J. Sel. Areas Commun..

[7] Alwakeel, A., Alnaim, A. & Fernandez, E. A survey of network function virtualization security. In *Proceedings of IEEE SoutheastConference* 1–8 (2018).

[8] Yu Z, Gao H, Wang D, Alnuaim A, Firdausi M, Mostafa A (2022). SEI^2^RS malware propagation model considering two infection rates in cyber-physical systems. Phys. A.

[9] Gong S, Chen J, Huang C, Zhu Q, Zhao S (2016). Virtual network embedding through security risk awareness and optimization. KSII Trans. Internet Inf. Syst..

[10] Zhang P, Li H, Ni Y, Gong F, Li M, Wang F (2020). Security aware virtual network embedding algorithm using information entropy TOPSIS. J. Netw. Syst. Manag..

[11] Liu S, Cai Z, Xu H, Xu M (2015). Towards security-aware virtual network embedding. Comput. Netw..

[12] Qu L, Assi C, Shaban K (2016). Delay-aware scheduling and resource optimization with network function virtualization. IEEE Trans. Commun..

[13] Pham C, Tran N, Ren S, Saad W, Hong C (2020). Traffic-aware and energy-efficient vNF placement for service chaining: Joint sampling and matching approach. IEEE Trans. Serv. Comput..

[14] Soualah, O., Mechtri, M., Ghribi, C. & Zeghlache, D. Energy efficient algorithm for VNF placement and chaining. In *Proceedings of IEEE/ACM International Symposium on Cluster, Cloud and Grid Computing*, *CCGRID* 579–588 (2017).

[15] Vidal I, Nogales B, Lopez D, Rodriguez J, Valera F, Azcorra A (2021). A secure link-layer connectivity platform for multi-site NFV services. Electronics.

[16] Liu X, Wang B, Liu S, Yang Z, Zhao Z (2018). Heuristic algorithm for secure virtual network embedding. Syst. Eng. Electron..

[17] Zhang P, Wang C, Jiang C, Benslimane A (2021). Security-aware virtual network embedding algorithm based on reinforcement learning. IEEE Trans. Netw. Sci. Eng..

[18] Firoozjaei M, Jeong J, Ko H, Kim H (2017). Security challenges with network functions virtualization. Future Gener. Comput. Syst..

[19] Fysarakis, K., Petroulakis, N., Roos, A., Abbasi, K., Vizarreta, P., Petropoulos, G., Sakic, E., Spanoudakis, G. & Askoxylakis, I. A reactive security framework for operational wind parks using service function chaining. In *Proceedings of IEEE Symposium on Computers and Communications*, *ISCC* 663–668 (2017).

[20] Rebello, G., Alvarenga, I., Sanz, I. & Duarte, O. BSec-NFVO: A blockchain-based security for network function virtualization orchestration. In *Proceedings of 2019 IEEE International Conference on Communications*. 1–6 (2019).

[21] Rashidi B, Fung C, Bertino E (2017). A collaborative DDoS defence framework using network function virtualization. IEEE Trans. Inf. Forens. Secur..

[22] Alhebaishi, N., Wang, L. & Jajodia, S. Modeling and mitigating security threats in network functions virtualization (NFV). In *Proceedings of 34th Annual IFIPWG Conference* 3–23 (2020).

[23] Zhao D, Luo L, Yu H, Chang V, Buyya R, Sun G (2021). Security-SLA-guaranteed service function chain deployment in cloud-fog computing networks. Clust. Comput..

[24] Tseng M, Tran T, Ha H, Bui T, Lim M (2021). Sustainable industrial and operation engineering trends and challenges Toward Industry 4.0: A data driven analysis. J. Ind. Prod. Eng..

[25] Xie Y, Wang S, Dai Y (2020). Revenue-maximizing virtualized network function chain placement in dynamic environment. Future Gener. Comput. Syst..

[26] Qi D, Shen S, Wang G (2019). Towards an efficient VNF placement in network function virtualization. Comput. Commun..

[27] Qu L, Assi C, Khabbaz M, Ye Y (2020). Reliability-aware service function chaining with function decomposition and multipath routing. IEEE Trans. Netw. Serv. Manag..

[28] Tang L, Zhao G, Wang C, Zhao P, Chen Q (2018). Queue-aware reliable embedding algorithm for 5G network slicing. Comput. Netw..

[29] Zhao D, Ren J, Lin R, Xu S, Chang V (2019). On orchestrating service function chains in 5G mobile network. IEEE Access.

[30] Han X, Meng X, Yu Z, Kang Q, Zhao Y (2020). A service function chain deployment method based on network flow theory for load balance in operator networks. IEEE Access.

[31] Li D, Hong P, Xue K, Pei J (2018). Virtual network function placement considering resource optimization and SFC requests in cloud datacenter. IEEE Trans. Parallel Distrib. Syst..

[32] Pei J, Hong P, Xue K, Li D (2019). Efficiently embedding service function chains with dynamic virtual network function placement in geo-distributed cloud system. IEEE Trans. Parallel Distrib. Syst..

[33] Hawilo H, Jammal M, Shami A (2019). Network function virtualization-aware orchestrator for service function chaining placement in the cloud. IEEE J. Sel. Areas Commun..

[34] Liu X, Wang B, Yang Z (2018). Virtual network embedding based on topology potential. Entropy.

[35] Su Y, Meng X, Zhao Z, Li Z (2019). Cognitive virtual network embedding algorithm based on weighted relative entropy. KSII Trans. Internet Inf. Syst..

[36] Yu, Z., Sohail, A., Nofal, T. & Tavares, J. Explainability of neural network clustering in interpreting the COVID-19 emergency data. *Fractals*. **30**(5), 2240122 (2022).

[37] Yu, Z., Arif, R., Fahmy, A. & Sohail, A. Self organizing maps for the parametric analysis of COVID-19 SEIRS delayed model. *Chaos Solitons & Fractals*. **150**, 111202 (2021). 10.1016/j.chaos.2021.111202 PMC8221985 34188365

[38] Yu, Z., Ellahi, R., Nutini, A., Sohail, A. & Sait, S. Modeling and simulations of CoViD-19 molecular mechanism induced by cytokines storm during SARS-CoV2 infection. *Journal of Molecular Liquids*. **327**, 114863 (2021). 10.1016/j.molliq.2020.114863 PMC7698669 33281252

